# OptForce: An Optimization Procedure for Identifying All Genetic Manipulations Leading to Targeted Overproductions

**DOI:** 10.1371/journal.pcbi.1000744

**Published:** 2010-04-15

**Authors:** Sridhar Ranganathan, Patrick F. Suthers, Costas D. Maranas

**Affiliations:** 1 Huck Institutes of Life Sciences, The Pennsylvania State University, University Park, Pennsylvania, United States of America; 2 Department of Chemical Engineering, The Pennsylvania State University, University Park, Pennsylvania, United States of America; University of Illinois at Urbana-Champaign, United States of America

## Abstract

Computational procedures for predicting metabolic interventions leading to the overproduction of biochemicals in microbial strains are widely in use. However, these methods rely on surrogate biological objectives (e.g., maximize growth rate or minimize metabolic adjustments) and do not make use of flux measurements often available for the wild-type strain. In this work, we introduce the OptForce procedure that identifies all possible engineering interventions by classifying reactions in the metabolic model depending upon whether their flux values must increase, decrease or become equal to zero to meet a pre-specified overproduction target. We hierarchically apply this classification rule for pairs, triples, quadruples, etc. of reactions. This leads to the identification of a sufficient and non-redundant set of fluxes that *must* change (i.e., MUST set) to meet a pre-specified overproduction target. Starting with this set we subsequently extract a minimal set of fluxes that must actively be *forced* through genetic manipulations (i.e., FORCE set) to ensure that all fluxes in the network are consistent with the overproduction objective. We demonstrate our OptForce framework for succinate production in *Escherichia coli* using the most recent *in silico E. coli* model, *i*AF1260. The method not only recapitulates existing engineering strategies but also reveals non-intuitive ones that boost succinate production by performing coordinated changes on pathways distant from the last steps of succinate synthesis.

## Author Summary

Over the past few years, there has been an unprecedented increase in the use of microorganisms for the production of biofuels, industrial chemicals and pharmaceutical precursors. In this regard, biotechnologists are confronted with the challenge to efficiently convert biomass and other renewable resources into useful biochemicals. With the advent of organism-specific mathematical models of metabolism, scientists have used computations to identify genetic modifications that maximize the yield of a desired product. In this paper, we introduce OptForce, an algorithm that identifies all possible metabolic interventions that lead to the overproduction of a biochemical of interest. Unlike existing techniques, OptForce does not rely on the maximization of a fitness function to predict metabolic fluxes. Instead, OptForce contrasts the metabolic flux patterns observed in an initial strain and a strain overproducing the chemical at the target yield. The essence of this procedure is the identification of all coordinated reaction modifications that force the network towards the overproduction target. We used OptForce to predict metabolic interventions for succinate overproduction in *Escherichia coli*. The results described in this paper not only uncover existing strain designs for succinate production but also elucidate new ones that can be experimentally explored.

## Introduction

An overarching challenge for metabolic engineers is to optimize the conversion of biomass and other renewable resources into useful metabolic products through fermentation and other biological conversions [1], [2]. Metabolic reaction fluxes are a fundamental determinant of the cell physiology, primarily because they provide a degree of engagement of various pathways in metabolic processes [3]. Earlier efforts addressed parts of metabolism with an emphasis on dynamics using kinetic approximations of reaction rates [4]–[7]. These approximations included the popular the S-system representation [4], [8]–[12] and Michaelis-Menten based descriptions [13]–[15]. Despite many success stories, it is increasingly becoming accepted that strain optimization requires taking account of the totality of biotransformations present in a production strain. This global view of metabolism is needed to enable the complete elucidation of all carbon fluxes diverted away from the desired product, diagnose unbalanced cofactor requirements limiting the extent of reactions as well as remedy deficiencies in the production of all biomass components leading to growth arrest.

Flux balance analysis (FBA) has emerged as an important framework [16]–[19] to assess the metabolic potential of a microbial production system. By taking a complete inventory of all (known) metabolic capabilities of an organism, FBA can assess the maximum possible yield of a desired product for different substrates and growth levels [20]. Given the lack of a truly predictive nature, FBA results must be carefully interpreted as performance limits and supplemented with MFA data whenever possible. Shortly after the introduction of FBA, a number of computational tools emerged that identified strain engineering modifications leading to targeted overproductions. One of the earliest efforts was the OptKnock [21] procedure that suggested gene knockouts leading to targeted overproductions. A bilevel optimization framework was postulated that computationally coupled the desired overproduction target to growth with unforeseen, at the time, implications for strain stability. Later, OptReg [22] extended OptKnock to consider not only knockouts but also overexpressions and down regulations of various reactions in the network. In addition, OptStrain [23] allowed for knock-ins of non-native functionalities from a comprehensive universal database of reactions to enable production of desired biochemicals. Evolutionary search procedures for solving the resulting combinatorial optimization problems were explored in OptGene [24] and applied for the production of succinic acid, glycerol and vanillin in yeast. The Ensemble Modeling approach [25] circumvented the kinetic modeling approach by incorporating flux measurements from knockout and enzyme overexpression experiments. Recently, the GDLS algorithm [26] was used for reduced metabolic models employing GPR associations to predict gene knockouts for succinate and acetate production in *E. coli*. So far, computational strain design procedures have been applied for a variety of metabolic engineering projects including the overproduction of lactic acid [21], [27], succinate [24], [28]–[31], 1,3-propanediol [21], hydrogen [23], amino acids [32], L-lysine [33], L-valine [34], threonine [35], lycopene [36], [37], ethanol in *E. coli* [22], [38], [39] and *Saccharomyces cerevisiae* [40] and bioelectricity in *Geobacter sulfurreducens* [41].

The use of computational tools operating on metabolic reconstructions to identify strain modifications is becoming commonplace. Nevertheless, a number of shortcomings plague all existing approaches. All are sequential in nature generating a single engineering strategy per run thus requiring multiple restarts to generate a set of candidate list of alternatives (i.e., typically less than ten) that is dwarfed by the myriads of engineering possibilities afforded by genome-scale models spanning thousands of reactions. Furthermore, in the absence of kinetic descriptions OptKnock and other methods rely on the maximization of surrogate biological fitness functions (e.g. maximization of biomass yield [21] or minimization of metabolic adjustments MOMA [42]) to estimate flux redirection upon strain engineering. These estimates may or may not be an accurate representation of how metabolism responds to genetic or environmental perturbations with significant consequences in the quality of the suggested re-designs. Existing methods do not pro-actively make use of flux measurements for the wild-type and/or an engineered strain to identify which fluxes need to be actively engineered in response to a production target. To remedy these limitations, we introduce a new computational framework termed OptForce that identifies all possible engineering interventions for a wild-type strain characterized by specific metabolic flux data consistent with an imposed production target(s).

## Methods

### Computing the flux variability for the wild-type and overproducing networks

The key concept of OptForce is to maximally resolve which fluxes (or combinations thereof) must depart away from the range of values allowed to span in the wild-type strain in response to an overproduction target. This maximal range of flux variability for the wild-type strain can be elucidated by iteratively maximizing and minimizing each flux [20], [43] subject to the stoichiometric constraints, uptake conditions and MFA flux data (either exact values or ranges) whenever available for the wild-type strain. This yields a set of lower and upper bounds for every flux in the metabolic network. Narrow ranges for the bounds are indicative of fluxes whose value is well bracketed given the information available for the wild-type strain whereas wide ranges indicate fluxes that are not significantly limited by the imposed (stoichiometric, MFA, etc.) constraints. Flux ranges can be used not only for characterizing the metabolic flux limits of the wild-type strain but also for identifying all flux combinations consistent with a single (i.e., v>v*^target^*) or multiple desired overproduction targets (see Appendix A of Text S1 for optimization formulations). The flux ranges consistent with the overproduction target(s) can be derived as before by iteratively maximizing and minimizing every flux in the metabolic network subject to stoichiometric constraints, uptake conditions and overproduction targets.

### Identifying the necessary changes in the network for overproduction (MUST sets)

**Figure 1:**
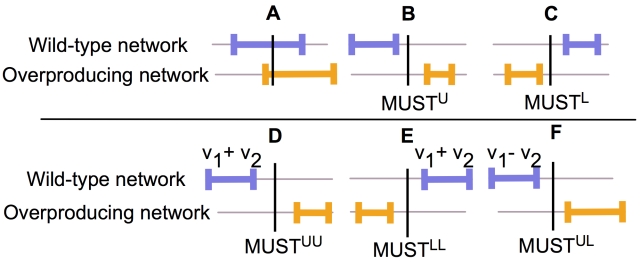
Maximal flux variability for the wild-type (blue) and overproducing (yellow) metabolic networks.

Contrasting the flux ranges for the (wild-type) metabolic network against the ones consistent with the overproduction target(s) provides the cornerstone of OptForce. Figure 1 pictorially illustrates the proposed concept. By superimposing the flux ranges for a given reaction in the wild-type vs. the overproducing network a number of possible outcomes are revealed. If there is any degree of overlap between the two reaction flux ranges (Figure 1a) then it may be possible to achieve the overproduction target without changing the value of the corresponding reaction flux in the wild-type strain. In contrast, if the flux ranges for a reaction in the wild-type metabolic network are completely to the left (Figure 1b) or to the right (Figure 1c) of the corresponding ranges for the overproducing metabolic network then the overproduction target cannot be achieved unless the reaction flux is directly or indirectly changed. The case depicted in Figure 1b calls for an increase whereas the one shown in Figure 1c requires a decrease in the reaction flux value. Note that if the reaction flux range collapses to zero then the corresponding reaction needs to be eliminated (e.g., through a gene knock-out). The gap between the two flux ranges quantifies the degree of required reaction flux modification. This reaction flux modification does not necessarily have to be realized by actively engineering the gene that codes for the enzyme catalyzing the reaction (e.g., through changed promoter, codon usage, or gene disruption/knock-out). It may come about indirectly by propagating through stoichiometry the effect of modifications occurring in other parts of metabolism (e.g., coupled reactions in series, cofactor coupling, etc.).

We refer to reaction fluxes that must increase (see Figure 1b) in the face of the imposed overproduction requirements as MUST^U^ whereas the ones that must decrease (see Figure 1c) as MUST^L^. Fluxes of reactions with overlapping ranges (see Figure 1a) between the wild-type and overproducing network do not provide any imperatives on network modifications when considered one at a time. Therefore, we further scrutinize them by considering sums of two reaction fluxes at a time and subsequently calculating their ranges in the wild-type and overproducing metabolic networks. This concept is similar to the use of residue doubles in the dead-end elimination algorithm for protein design [44]. As was the case of single reaction fluxes, three outcomes are possible (see Figure 1d–f). Non-overlapping ranges imply that in the overproducing network *either one or the other reaction flux* (but not necessarily both) must increase (Figure 1d) or decrease (Figure 1e) in value. These pairs of reactions form sets MUST^UU^ and MUST^LL^ respectively. One can extend this concept further by analyzing the range of not just the sum of two fluxes but also their difference for the wild-type and overproducing networks (see Figure 1f). As before, non-overlapping ranges imply that *either the first reaction flux must increase or the second reaction flux must decrease*. By extension, these pairs of reactions form the equivalent sets MUST^UL^ and MUST^LU^, respectively. One can systematically extend this analysis by considering sums and/or differences of three, four, etc. reactions at a time. Collectively, the derived sets (e.g., MUST^L^, MUST^U^, MUST^UU^, MUST^LLL^, MUST^UULL^, etc.) encompass all the necessary reaction flux changes that MUST take place in the wild-type metabolic network for the desired overproduction. Appendix B in Text S2 introduces a bilevel formulation for identifying all MUST sets without relying on exhaustive enumerations inspired by a similar representation introduced earlier [45] for identifying synthetic lethal deletions.

### Identifying the minimal set of engineering interventions (FORCE sets)

The next step of OptForce is to identify how the collective set of changes (encoded within the MUST sets) can be imparted on the wild-type metabolic network with the minimal number of direct interventions (i.e., knock-up/down/outs). The identified MUST sets encode Boolean choices regarding which fluxes (or combinations thereof) must change in value. Upon the incorporation of these constraints, an optimization formulation is proposed (see Appendix C in Text S3) that finds the minimum number of imparted changes (through gene knock-outs/up/downs) so as the overproducing metabolic network involves no feasible metabolic phenotypes that fail to meet the imposed production target. The collective set of minimal network modifications that yield the desired overproduction target is referred to as the FORCE set and is typically represented as a Boolean diagram globally depicting all minimal alterative choices for engineering the wild-type network. Many of the reactions in the FORCE set are also members of various MUST sets.

The optimization formulations for computing the allowable flux values for all reactions in the wild-type metabolic network are provided in Appendix A (see Text S1). The derivation and solution procedure of bilevel optimization formulations for exhaustively elucidating the membership in the MUST sets are provided in Appendix B (see Text S2). The bilevel optimization formulation for identifying the FORCE set of engineering interventions is given in Appendix C (see Text S3). All optimization problems were solved using the GAMS/CPLEX (version 9.1) solver on a 2.6 GHz AMD Opteron Processor with 32 GB of ECC RAM.

## Results

In this section, we benchmark the OptForce framework by identifying metabolic interventions that lead to the overproduction of succinate using the latest genome-scale metabolic model for *E. coli*, *i*AF1260 [46]. There have been extensive efforts to re-engineer metabolic pathways in *E. coli* for improving succinate yield [31], [47]–[62]. We explored the production of succinate under anaerobic conditions to take advantage of the inherently high yield towards succinate [55]. Under anaerobic conditions, the synthesis route for succinate takes place along the reductive arm of the TCA cycle and involves the conversion of oxaloacetate (OAA) to malate, fumarate and eventually to succinate. The initial strain was characterized by estimating the maximal range of flux variability using intracellular flux measurements available for the wild-type strain of *E. coli*, MG1655 [61]. The OptForce algorithm was used to explore engineering interventions under three different scenarios. First, we identified strain modifications that guarantee 100% theoretical yield for succinate. Not surprisingly, these engineering modifications come at the expense of completely negating biomass formation. Therefore, we next examined the difference in the obtained results when imposing a secondary performance target for biomass formation at or above 1% of its theoretical yield. In the third case study, we examined the effect of adding the activity of the heterologous pyruvate carboxylase (*pyc*) gene to the *i*AF1260 model of *E. coli*. Note that the abbreviations and directionalities of reactions adhere to the *i*AF1260 metabolic model definitions.

### Case 1: Succinate overproduction target at 100% of its theoretical maximum yield

**Figure 2:**
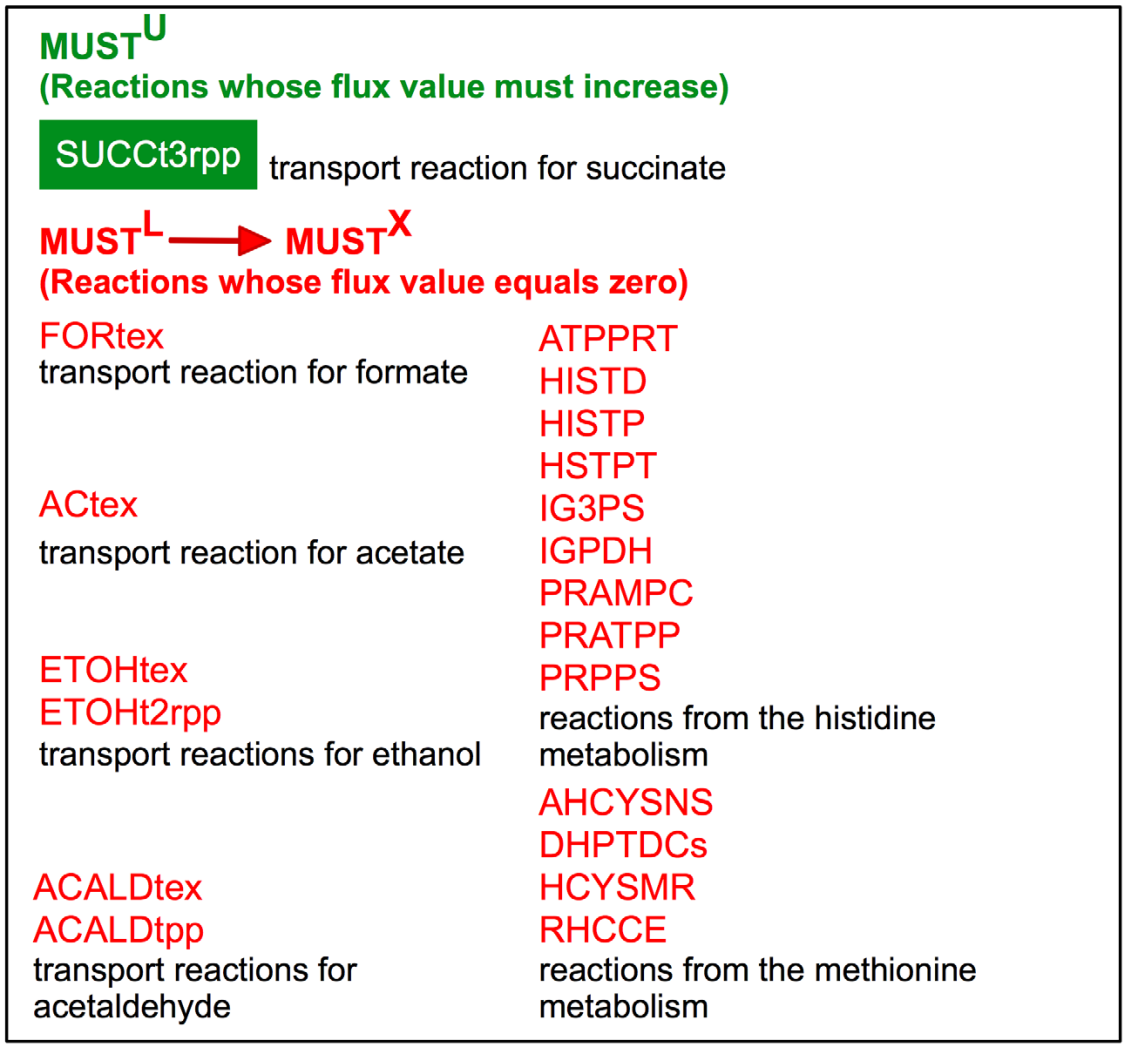
MUST^U^ and MUST^X^ set of reactions identified by OptForce for 100% theoretical yield of succinate.

Figure 2 lists the identified MUST^U^ and MUST^L^ sets of reactions whose fluxes must depart the original ranges. Note that because all members of set MUST^L^ involve fluxes set to zero we re-designate them as MUST^X^ to signify that they all correspond to reaction eliminations. Not surprisingly, the transport reaction directing succinate out of the cytosol (SUCCt3rpp) was classified into the MUST^U^ whereas transport reactions for competing by-products such as ethanol (ETOHt2rpp, ETOHtex), acetate (ACtex), formate (FORtex) and acetaldehyde (ACALtpp, ACALDtex) were completely blocked (i.e., members of the MUST^X^ set). In addition, a number of reactions from hisitidine (ATPPRT, HISTD, HISTP, HSTPT, IG3PS, IGPDH, PRAMPC, PRATPP and PRPPS) and methionine metabolism (AHCYSNS, DHPTDCs, HCYSMT and RHCCE) were also set to zero. Note that these reactions are essential for amino acid biosynthesis and are fully coupled to growth. Therefore, the drain of carbon flux from the pentose phosphate pathway towards histidine and methionine synthesis is prevented thus halting the production of biomass.

**Figure 3:**
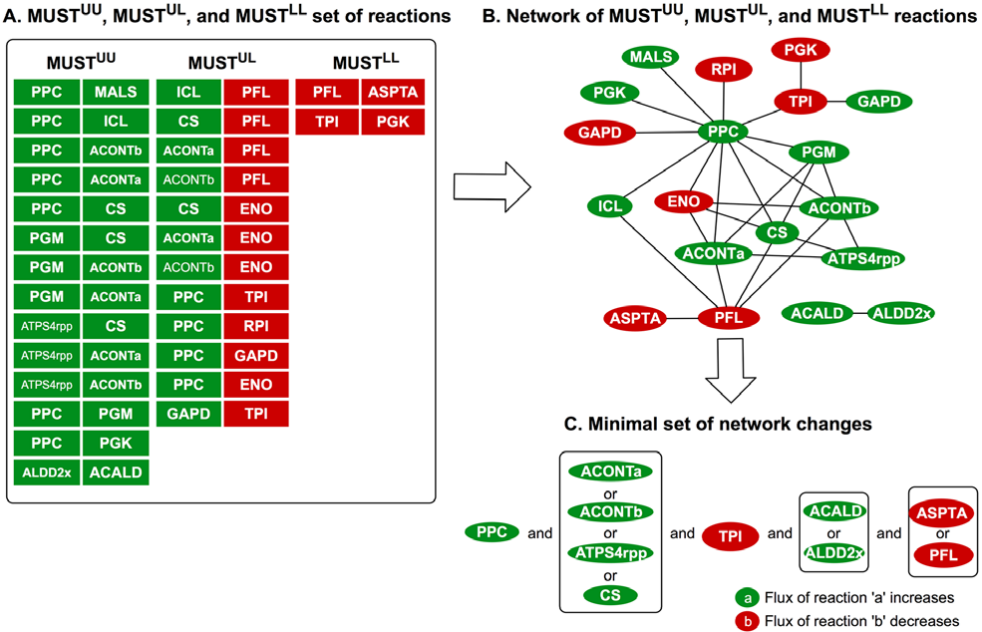
MUST^UU^, MUST^UL^, and MUST^LL^ set of reactions. Figure 3a shows the list of reaction pairs in the MUST sets. Figure 3b shows the network of interacting reactions formed the list of all reaction pairs from Figure 3a. Reactions in green ovals indicate that its flux increases and red ovals indicate the decrease in flux values. Figure 3c represents the minimal set of network changes identified using Boolean logic that together span the entire network shown in Figure 3b.

While results for MUST^U^ and MUST^L^ involve primarily intuitive negations of by-products formation, sets MUST^UU^, MUST^UL^ and MUST^LL^ allude to more complex flux re-allocations (see Figure 3). For example, in the MUST^UU^ set the increase in the flux for reaction phosphoenolpyruvate carboxylase (PPC) can only be compensated by the simultaneous increase in the flux of five TCA cycle reactions (i.e., MALS, CS, ACONTa, ACONTb and ICL). This implies that at least one of two possible avenues for succinate production must be increased under anaerobic conditions (see Figure 3a). Specifically, either the flux along the traditional succinate synthesis route through the reductive pathway that converts oxaloacetate (oaa) to malate and fumarate or the flux through the glyoxylate shunt needs to increase. Interestingly, the higher succinate yield of the latter mechanism due to NADH availability has been implemented in *E. coli* by deactivating the *iclR* repressor (to activate the glyoxylate bypass) under anaerobic conditions by [59].

Figure 3a reveals that a number of flux up-regulations (e.g., PPC, PGM, CS, ICL, ACONTa/b, PGM, ATPS4rpp, ALDD2x, ACALD) and down-regulations (e.g., PFL, TPI, RPI, ASPTA, PGK) appear frequently as choices in multiple pairs. These mutually compensatory flux changes can be more clearly discerned by fusing all interacting components from MUST^UU^, MUST^UL^ and MUST^LL^ into a single graph (see Figure 3b) where fluxes that increase are shown in green and those that decrease are shown in red. The importance of PPC up-regulation is manifested by the fact that as many as ten separate reaction flux modifications would be needed to replace it. Similarly, the decrease in flux through PFL can only be compensated by up-regulating the flux of four reactions along the glyoxylate shunt while the down-regulation of the flux through ENO can only be replaced by the up-regulation of four reactions supplying flux to the TCA cycle. The compensatory interconnections in Figure 3b suggest that not all depicted flux modifications are simultaneously needed to reach the desired phenotype (i.e., 100% yield of succinate). Instead, all flux modifications implied by sets MUST^LL^, MUST^UU^ and MUST^UL^ can be satisfied by up- or down-regulating a minimal set of reactions. We identified all such minimal reaction flux modification sets and depicted them in the form of a Boolean diagram in Figure 3c. As expected, up-regulation of the flux through PPC is a consensus choice while the up-regulation of only one out of ACONTa, ACONTb, CS and ATPS4rpp is needed. Interestingly, the down-regulation of PFL which diverts flux towards organic acids such as formate, lactate, acetate, ethanol, etc. emerged as a required change despite its relatively low connectivity in the diagram of Figure 3b.

**Figure 4:**
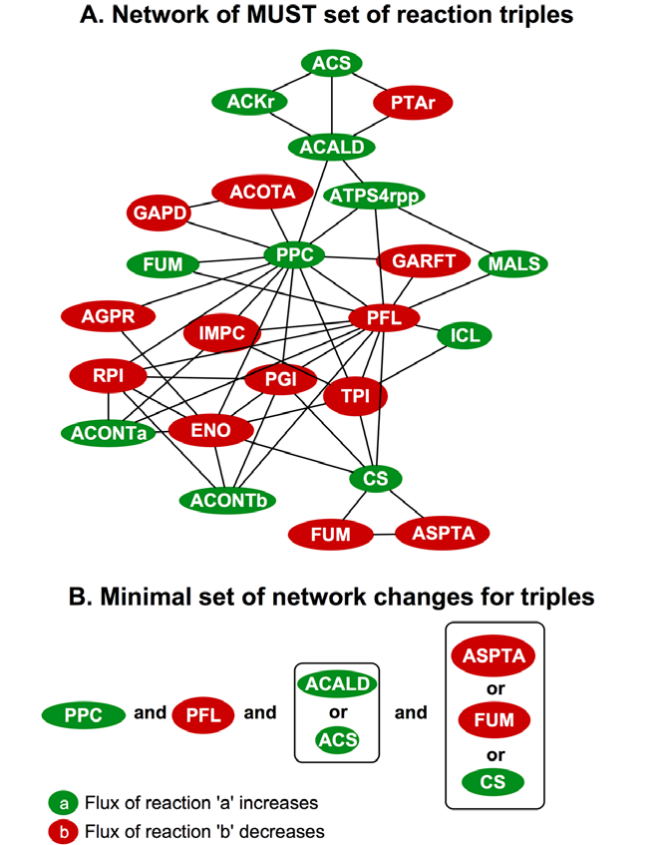
MUST^UUU^, MUST^UUL^, MUST^ULL^ and MUST^LLL^ set of reactions. Network of all the interacting components (Figure 4a) and the minimal set of network modifications (Figure 4b) for reactions in the MUST^UUU^, MUST^UUL^, MUST^ULL^ and MUST^LLL^ sets.

Figure 4 depicts the reaction flux modifications needed when considering three reaction fluxes at a time (one out of three). The reactions are denoted as ovals where green nodes represent the flux of the reaction that increases and red nodes indicate those that decrease. They span up-regulations (MUST^UUU^), down-regulations (MUST^LLL^) or combinations thereof (MUST^UUL^ and MUST^ULL^). Figure 4a re-affirms the key role of up-regulating PPC but also reveals the importance of redirecting the flux of reactions from pyruvate metabolism (i.e. PFL, ACS, ACALD, ACKr, PTAr) towards acetyl-CoA. Furthermore, Figure 4a reveals that the decrease in the value of the flux for phosphotransacetylase (PTAr) and acetate kinase (ACKr) reduces the export of acetate and increases the amount of acetyl-CoA available for the glyoxylate pathway. These results are in agreement with the knockouts for *ackA* and *pta* in strain SBS990MG constructed for succinate synthesis [59]. The reaction modifications implied in MUST^LLL^, MUST^UUU^, MUST^UUL^ and MUST^ULL^ can also be distilled into a minimal set of modifications (see Figure 4b). Many of these modifications were present in Figure 3c, however, a number of new imperatives such as reducing the flux of FUM emerge. One can methodically, continue to identify additional constraints that need to be satisfied to achieve the desired phenotype by looking into higher-order combinations of fluxes. The results for reactions quadruples are provided as supplementary material (see supporting information - Text S4 and Figure S1).

**Figure 5:**
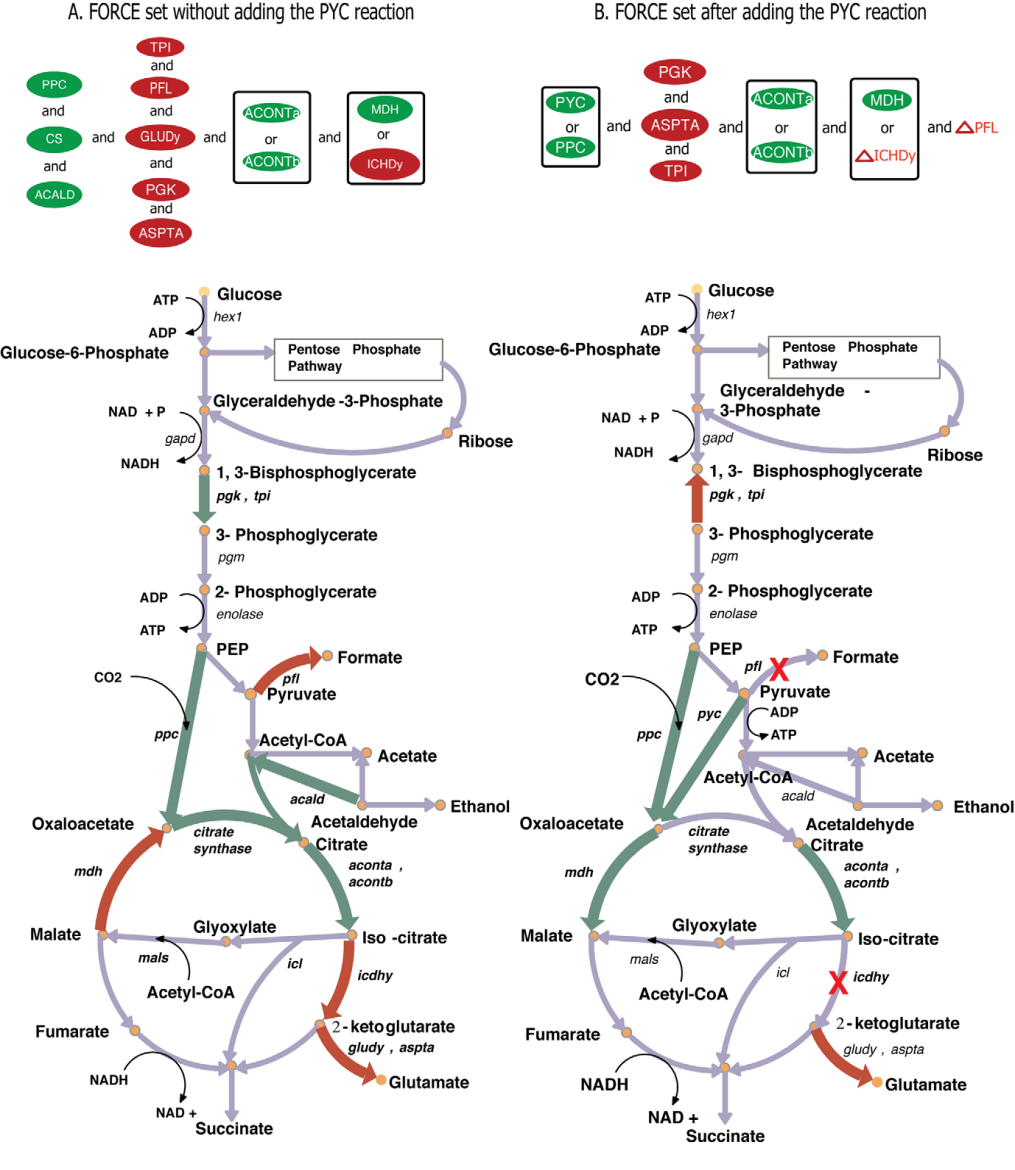
FORCE set of reactions for succinate overproduction on a metabolic map of *E. coli*. Figure 5a shows the interventions for cases 1 and 2 before adding the PYC reaction and Figure 5b shows the interventions after adding the PYC reaction. Reaction names shown in green ovals indicate the FORCE set whose fluxes must be increased while the red ones indicate the ones that must be decreased. Reaction names adjacent to small red triangles represent knockouts.

We next used the bilevel optimization formulation (refer Appendix C in Text S3) to identify the minimal set of reaction modifications (i.e., FORCE set) that guarantee the imposed yield (100% succinate yield). Note that the identified MUST reaction flux modifications were added as constraints in the FORCE set formulation. However, we found that the flux restraints (single, double and triple reaction combinations) in the MUST sets were insufficient to guarantee the target yield for succinate (i.e., min V_succinate_ = 64% of theoretical). This suggested that additional reactions that participate in higher-order (unexplored) MUST sets were required to guarantee the target yield for succinate. Upon allowing reactions absent from the MUST sets to become members of the FORCE set the imposed target for succinate production was met. The identified minimal set of forced modifications (see Figure 5a) is comprised of ten different interventions. The up-regulation of PPC and CS ensures that the pool of oxaloacetate is diverted towards the TCA cycle. The up-regulation for PGK and TPI increases the glycolytic activity providing precursor metabolites such as phosphoenol pyruvate, oxaloacetate etc. to succinate synthesis. The down-regulation of PFL, GLUDy and ASPTA prevents the formation of by-products such as formate, lactate, ethanol, glutamate, aspartate and 2-ketoglutarate. The up-regulation for ACALD converts any residual acetate back into acetyl-CoA, which in turn is converted to succinate. Notably, for two such interventions there exist two equivalent alternatives. The first one involves the up-regulation of either of ACONTa/b isozymes to ensure conversion of citrate into glyoxylate and succinate. The second one requires either the down-regulation of malate dehydrogenase (MDH) that converts malate into oxaloacetate or the down-regulation of ICDHy that diverts flux away from the glyoxylate shunt. Interestingly, none of the transport reaction regulations identified in the MUST^U^ and MUST^X^ sets are present in the FORCE sets. The optimization formulation for the FORCE set identified more economical upstream flux modifications that negated the formation of multiple by-products. A consequence of imposing 100% yield to succinate is that biomass formation is halted as histidine and methionine formation is seized. In the next section, we examine how the identified engineering interventions change when a 1% biomass requirement is imposed simultaneously with a 98% yield requirement for succinate. In addition, we contrast the magnitude of the imposed flux changes for the two different scenarios.

### Case 2: Succinate overproduction target at 98% theoretical yield while allowing for 1% yield of biomass

Figure S2 (see supplementary information) lists all MUST sets involving single, double and triple reaction combinations. As expected, we find that by dialing back the requirement for succinate production the number of flux modifications that must happen in the network to meet the new requirement is reduced. Lowering the yield of succinate from 100 to 98% eliminates all reaction deletions (i.e., members of the MUST^X^ set) belonging to competing pathways. The ethanol transport reactions (ALCD2x and ETOHt2rpp) do not have to be completely eliminated but rather lowered in value to 3 mmol/gDW.hr from a wild-type flux value of 19 mmol/gDW.hr.

**Figure 6:**
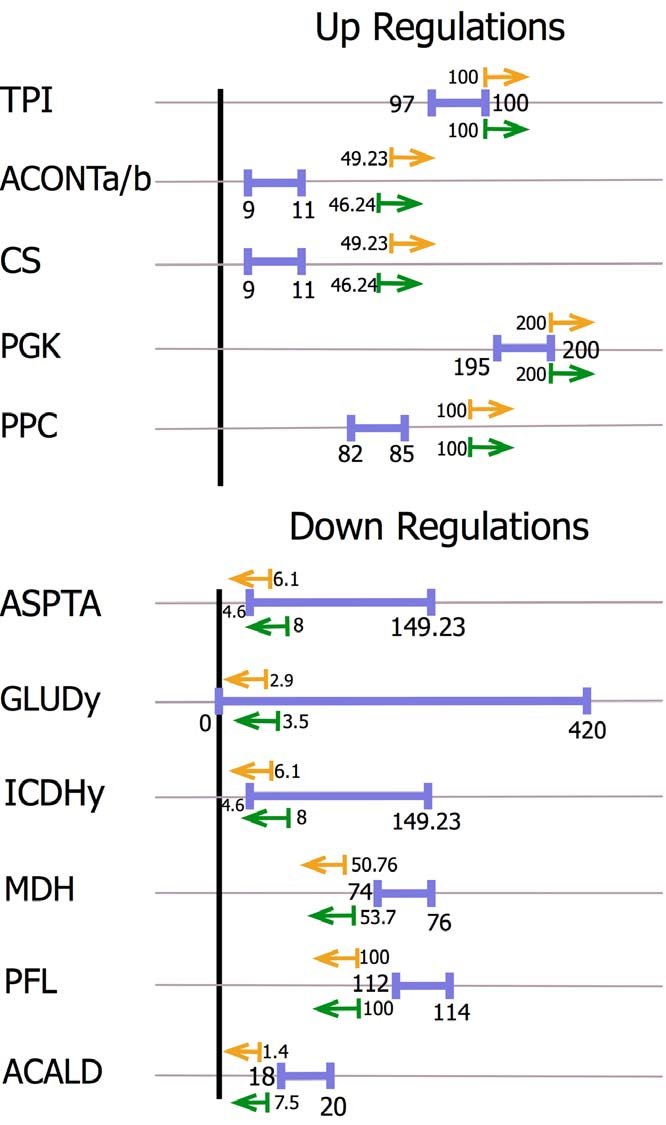
Comparison of the flux ranges for reactions in the FORCE sets. Blue lines indicate the wild-type flux ranges. The orange (case 1) and green (case 2) lines indicate the flux values beyond which these reactions must be engineered to guarantee the overproduction of succinate.

Despite the differences in the MUST sets between cases 1 and 2 the corresponding FORCE sets of reactions were identical. Up-regulations for PPC, CS, MALS, ICL and ACONTa and down regulations for reactions along the pathways leading to competing by-products were required for the 98% succinate yield case. Even though the membership of the FORCE set is the same the corresponding required levels of up or down-regulation are slightly different. Figure 6 depicts the original wild-type flux ranges and the new values that the reaction fluxes must reach to guarantee the imposed succinate production targets under cases 1 and 2 respectively. The largest difference between the two arises for the down-regulation of ACALD where a value of 7.5 mmol/gDW.hr suffices for case 2 while a value of 1.4 mmol/gDW.hr is needed for case 1. Note that a number of glycolytic fluxes are set at their stoichiometric upper bounds (i.e., PPC, PGK and TPI) implied by the uptake of 100 moles of glucose. Next, we explore how the addition of a single heterologous reaction (i.e., pyruvate carboxylase) radically changes the way that the network needs to be re-engineered.

### Case 3: Succinate overproduction upon the addition of pyruvate carboxylase

Pyruvate carboxylase (PYC) has been overexpressed in *E. coli* from *Lactococcus lactis* [58], [59] and *Rhizobium etli* [49]. The addition of the new reaction to the metabolic network boosts the succinate yield by 15.3% above the original theoretical maximum (1.72 moles/mole of glucose). PYC using ATP directly converts pyruvate into oxaloacetate which serves as a precursor for the glyoxylate and the fermentative pathway. In this study, we allowed the production of biomass at 1% of theoretical yield and identified the flux changes when succinate was produced at 98% of theoretical maximum (1.7 moles/mole of glucose).

**Figure 7:**
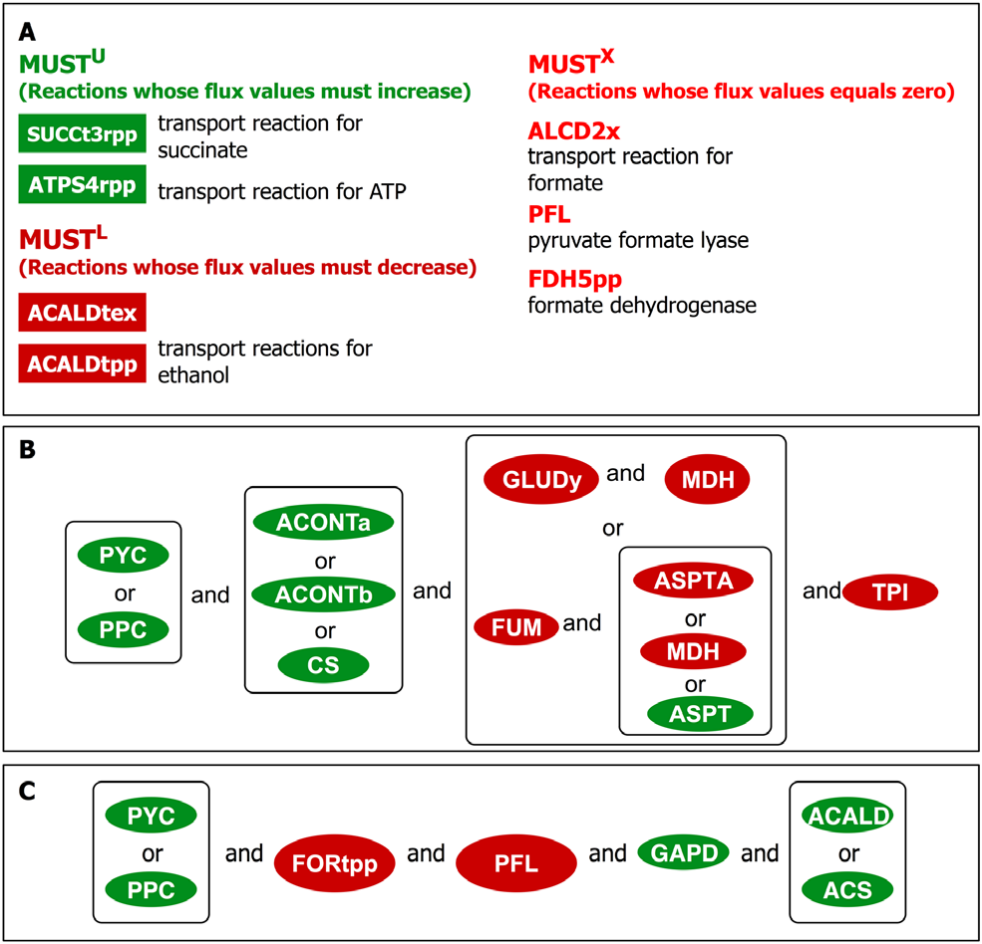
MUST set of reactions after the addition of the pyruvate carboxylase (PYC) reaction. Figure 7a shows the list of MUST^U^, MUST^L^ and MUST^X^ set of reactions. Figures 7b and 7c shows the minimal set of network modifications required for the doubles and triples, respectively, for case 3.

Figure 7 shows the results for the MUST set of reactions. As expected, the transport reaction for succinate and ATP are both members of the MUST^U^ set whereas the transport reaction for acetaldehyde is classified as MUST^L^. The required increase in the flux for ATP is due to the ATP consuming pyruvate carboxylase. Unlike cases 1 and 2, the synthesis route for by-products (formate and acetyl-CoA) consuming pyruvate through the pyruvate formate lyase (PFL), alcohol dehydrogenase (ALCD2x) and formate dehydrogenase (FDH5pp) reactions are completely shut off to afford a complete conversion of pyruvate to OAA. This suggests that the presence of PYC provides an alternative route to PPC whereby OAA can be replenished either by increasing the flux through PPC or PYC. This is in agreement with the experimental findings by Ka-Yiu San and coworkers [59] that a drop in the activity of one the two enzymes can be compensated by the other.

The FORCE set of engineering interventions for this scenario is contrasted against cases 1 and 2 and is shown in Figure 5b. The addition of the PYC reaction significantly reduces the number of engineering interventions required to guarantee the target yield for succinate. The interventions required to reduce the drain of carbon away from the pyruvate metabolism are absent indicating that the pyruvate carboxylase enzyme can safeguard against the consumption of pyruvate towards side-products. However, the down regulation for ASPTA is again needed to reduce the secretion of aspartate and glutamate. Importantly, the up-regulation for PYC could be substituted by up-regulating PPC which suggest that the OAA pool can be replenished by either of these two reactions. The increase in activity for some reactions in the glycolytic pathways (TPI, PGK) and the TCA cycle (ACONTa, ACONTb and MDH) is required as before. In contrast with the previous case-study, the complete elimination of PFL and isocitrate dehydrogenase (ICDHy), rather than just their down-regulation is needed. The elimination of PFL is imposed to completely prevent the conversion of pyruvate into by-products. The elimination of ICDHy blocks the flow of carbon flux through the TCA cycle into the glutamate pathway thus ensuring the complete conversion of isocitrate into glyoxylate and succinate.

## Discussion

In this paper, an optimization-based methodology called OptForce was introduced for predicting all possible metabolic modifications that could guarantee, subject to the model stoichiometry and conditions, a pre-specified overproduction level of a desired biochemical. The results for succinate overproduction in *E. coli* reveal that the needed interventions results remain the same upon requiring the production of a small amount of biomass but change significantly upon the addition of a key reaction to the model.

Many of the suggested interventions recapitulate existing strain redesign strategies for succinate synthesis. For example, experimental evidence suggests that the overexpression of PPC from *Sorghum vulgare* and *Actinobacillus succinogenes* in *E. coli* not only increases the yield of succinate but also reduces the secretion of acetate [31], [58], [59], [63]–[65]. In addition, succinate production has been enhanced by the increased carboxylation of PEP and pyruvate (to increase the pool of OAA for TCA cycle) in the *E. coli* mutant NZN111 by decreasing the activity for pyruvate formate lyase (PFL) and lactate dehydrogenase [47], [53]. Furthermore, Vemuri *et al.* [62], [66] made use of the glyoxylate pathway for succinate synthesis thus overcoming the limitation of NADH availability for the fermentation pathway. The up-regulations for the isozymes ACONTa/b and the down regulations for ICDHy, ASPTA and GLUDy predicted by OptForce allude to the same strategy of glyoxylate shunt utilization for succinate synthesis. Finally, multiple studies [31], [59]–[61] have shown that the deletion of *adhE* and *ackA-pta* coding for acetaldehyde dehydrogenase (ACALD) reduces the formation of by-products ethanol, acetate and acetaldehyde as suggested by OptForce.

The up-regulation of citrate synthase (CS), aconitase (ACONTa/b) and reactions from the glycolytic pathway (PGK and TPI) are engineering strategies suggested by OptForce that to the best of our knowledge have not yet been implemented for succinate production. Heterologous overexpression of the *citZ* gene from *Bacillus subtillis* that encodes citrate synthase increased the activity through the TCA cycle towards isocitrate and 2-ketoglutarate [67]. However, when this gene was overexpressed in *E. coli* strain SBS550MG, an increase in the yield of succinate was not observed [59]. The reason for this could be the absence of the down regulations for ICDHy and GLUDy that lead to the production of glutamate and other amino acids required for growth. The results predicted by OptForce suggest that by collectively incorporating the flux modulations for citrate synthase, isocitrate dehydrogenase and glutamate dehydrogenase along with the existing strategies, the yield of succinate can be further enhanced from the current experimental yield (1.7 moles/mole of glucose) as observed for strains SBS550MG and SBS990MG [59].

The genetic interventions predicted by OptForce underscore the importance of up-regulating key fluxes along the succinate pathway in addition to the knockouts for by-products. Existing strain optimization procedures (e.g. OptKnock [21] and OptReg [22]) that couple the maximization of growth rate and secretion of the product tend to prevent the yield of succinate from reaching the theoretical maximum. Table 1 contrasts the yields predicted for succinate overproduction by OptKnock [21], OptReg [22] and OptForce. OptKnock and OptReg rely on biomass maximization to perform flux allocation in the metabolic network whereas OptForce reports the most conservative value for succinate production allowed by the stoichiometry and conditions. It is noteworthy that for more than two interventions even the worst-case succinate yield predictions by OptForce are far more superior to strategies predicted by OptKnock and OptReg. Notably, OptForce suggested the down regulation but not the knockout of PFL and GLUDy [59] along with a number of additional interventions missed by both OptKnock and OptReg due to their inconsistency with biomass maximization.

**Table 1:** Comparison of the minimum guaranteed fluxes from OptKnock, OptReg and OptForce procedures for succinate production in *E. coli*.

	Results from OptKnock		Results from OptReg		Results from OptForce	
Number of metabolic interventions (K)	Knockouts	Minimum guaranteed flux for succinate ^(*)^ (mmol/gDW.hr)	Metabolic Interventions	Minimum guaranteed flux for succinate ^(*)^ (mmol/gDW.hr)	Metabolic Interventions from FORCE sets	Minimum guaranteed flux for succinate (mmol/gDW.hr)
K = 2	ALCD2x, GLUDy	5.5 (84.1)	PFL (×)PPC (↑)	2.1 (79.4)	PPC (↑), CS (↑)	84.6
	PFL, LDH	1.2 (76.8)	-	-	PPC (↑)MDH (↓)	50.8
K = 3	ALCD2x, PFL, LDH	5.9 (85.7)	PFL (×), PPC (↑), ALCD2x (↓)	2.8 (84.3)	PPC (↑), CS (↑)MDH (↓)	100.2
	ALCD2x, ACKr, PTAr	1.1 (84.6)	-	-	PPC (↑), ACONT (↑)MDH (↓)	100.2
K = 4	ALCD2x, ACKr, PTAr, PYK	4.9 (88.8)	PPC (↑), PDH (↓)ALCD2x(↓), CS(↑)	2.8 (88.4)	PPC (↑), CS (↑)PFL (↓), MDH (↓)	100.2
	ALCD2x, ACKr, PTAr, TKT1	2.1 (87.4)	-	-	PPC (↑), ACONT (↑)PFL (↓), MDH (↓)	100.2

^(*)^ The values within parentheses denote the maximum flux values for succinate from OptKnock and OptReg.

The OptForce procedure allows for the complete enumeration of engineering modifications consistent with an overproduction target(s). The incorporation of metabolic flux information about the wild-type network allows for a sharper elucidation of engineering interventions. The engineering interventions predicted by OptForce depend on the available flux measurements for the initial strain. OptForce can be modified to predict globally valid metabolic interventions by utilizing biological objectives (i.e. maximization of biomass) when sufficient metabolic flux data are not available. Furthermore, the procedure can hierarchically be applied at intermediate stages of a metabolic engineering project by re-calculating the set of engineering interventions as new flux data for (multiple) mutant strains become available. The restriction of minimality in the calculated FORCE set can be relaxed allowing for the exploration of less parsimonious engineering interventions. For example, we studied the case for identifying additional interventions after retaining the best eight out of the ten interventions originally identified by the OptForce method (for cases 1 and 2). However, we found that even after allowing seven additional interventions (i.e. K = 15), the resulting FORCE set was not sufficient to increase the yield to more than 80% of the theoretical maximum. In addition, reactions that cannot (e.g., diffusion limited transport, non-gene associated reactions, etc.) be directly manipulated can be excluded from consideration during the derivation of the FORCE set. It is to be noted that the OptForce procedure provides targets for genetic manipulations at the metabolic flux level. The lack of a completely quantitative mapping between gene expression and flux levels implies that multiple rounds of experimental strain modifications may be needed to translate the FORCE set of reaction fluxes to the required gene expression levels. An algorithmic implementation of the procedure is available as supplementary material (see supporting information - Text S5).

## Supporting Information

Text S1 Appendix A: Computing flux variability for the wild-type and overproducing networks
(0.09 MB DOC)

Text S2 Appendix B: Bilevel formulation for the identification of MUST sets
(0.05 MB DOC)

Text S3 Appendix C: Bilevel formulation for the identifying the FORCE set
(0.07 MB DOC)

Text S4 Results for MUST considered four-at-a-time (quadruples)
(0.12 MB DOC)

Text S5 Prototype Implementation for the OptForce Algorithm
(0.06 MB DOC)

Figure S1 Minimal set of network modifications for reaction quadruples.
(2.08 MB TIF)

Figure S2 MUST set of reactions for 98% yield of succinate. Figure S2a shows the list of reactions in the MUST^U^ and MUST^L^ sets. Figure S2b and S2c shows the network of interacting reactions and the minimal set of network modification for the doubles and triples, respectively, identified for case 2.
(3.34 MB TIF)
