# Supplementary material for: OptForce: An Optimization Procedure for Identifying All Genetic Manipulations Leading to Targeted Overproductions
Source: PLoS Comput Biol. 2010 Apr 15;6(4):e1000744. doi: 10.1371/journal.pcbi.1000744 (PMC2855329; doi:10.1371/journal.pcbi.1000744)
Supplement: Text S1 — Appendix A: Computing flux variability for the wild-type and overproducing networks (0.09 MB DOC) [file pcbi.1000744.s001.doc]

**OptFlux: An Optimization Procedure for Identifying All Genetic Manipulations Leading to Targeted Overproductions**

**Supporting Information: Text S1**

Sridhar Ranganathan^1^, Patrick F. Suthers^2^ and Costas D. Maranas^2^,*

**Appendix A: Computing flux variability for the wild-type and overproducing networks**

The following set, variable and parameter definitions are introduced to support the development of the optimization formulations needed for the derivation of the MUST and FORCE sets.

*Sets:*

N = {*i*} set of metabolites

M = {*j*} set of reactions

M­­^data^ = {*j*} subset of reactions with known flux values or ranges

M­­^uptakes^ = {*j*} subset of substrate transport reactions

M^targets^ = {*j*} subset of reactions targeted for overproduction

*Variables:*

*v_j_* flux through reaction *j*

*Parameters:*

*S_i,j_* stoichiometric coefficient of a metabolite *i* in a reaction *j*

measured flux ranges for a reaction *j* in the set M­­^data^

$v_{j}^{\text{uptake}}$ flux value for uptake reaction *j* in the set M­­^uptakes^

target flux values for all reactions within set M^targets^

Parameters $v_{j}^{\text{data},L}$ and $v_{j}^{\text{data},U}$ quantify range data available for a subset of reaction fluxes in the network. These data can be derived by directly measuring growth, uptake and/or secretion rates. In addition, MFA can provide information about many internal fluxes in the network either in the form of restraints or exact values. The use of data range parameters instead of fixed values allows us to make use of lower/upper bound data typically inferred from MFA data due to measurement error and other factors. Parameters $v_{j}^{\text{data},L}$ and $v_{j}^{\text{data},U}$ are set to the same value if an exact estimate is known for a particular flux.

Upon the incorporation of all available data encoded within parameters $v_{j}^{\text{data},L}$ and $v_{j}^{\text{data},U}$, the flux variability in the wild-type strain is derived by iteratively minimizing and maximizing each flux using a series of linear programming (LP) problems given by:

Constraints (2) impose stoichiometric balances on the network whereas constraints (3) incorporates all known flux data for reaction fluxes present in set M^data^. Constraints (4) set the uptake of carbon and other substrates and constraints (5) impose global upper and lower bounds for the remaining fluxes. The solution to this sequence of LPs yields the lower ($v_{j}^{\text{wt},L}$) and upper ($v_{j}^{\text{wt},U}$) flux range values for all reactions in the wild-type metabolic network. Reactions with tight flux ranges allude to direct or indirect coupling with the imposed wild-type network flux data whereas reactions with wide flux ranges tend to be largely insensitive to the availabe data. Parameters ($v_{j}^{\text{wt},L}$ and $v_{j}^{\text{wt},U}$) estimated from the above LPs represent the maximal flux variability for the wild-type metabolic network.

Similarly, the flux ranges $v_{j}^{\text{*,}L}$ and $v_{j}^{\text{*,}U}$ consistent with the imposed set of overproduction targets for all can be derived by iteratively solving (for every *j* in M) the following sequence of LP formulations:

This formulation does not include the constraint that imposes the flux data available for the wild-type network, instead through constraint (6) it sets the required production levels.
