# Supplementary material for: OptForce: An Optimization Procedure for Identifying All Genetic Manipulations Leading to Targeted Overproductions
Source: PLoS Comput Biol. 2010 Apr 15;6(4):e1000744. doi: 10.1371/journal.pcbi.1000744 (PMC2855329; doi:10.1371/journal.pcbi.1000744)
Supplement: Text S2 — Appendix B: Bilevel formulation for the identification of MUST sets (0.05 MB DOC) [file pcbi.1000744.s002.doc]

**OptFlux: An Optimization Procedure for Identifying All Genetic Manipulations Leading to Targeted Overproductions**

**Supporting Information: Text S2**

Sridhar Ranganathan^1^, Patrick F. Suthers^2^ and Costas D. Maranas^2^,*

**Appendix B: Bilevel formulation for the identification of MUST sets**

The identification of sets MUST^L^ and MUST^U^ is conducted by testing whether inequalities and respectively, are satisfied by exhaustively testing one at a time all reactions in the network. Second-order MUST sets such as MUST^LL^, MUST^UU^ and MUST^LU^ require exhaustively exploring all pairwise sums/differences of reactions which becomes computationally long running for genome-scale models due to the large number of LP problems that need to be solved. The elucidation of higher-order sets (e.g., MUST^LLL^, MUST^UUUU^, etc.) using a brute force exhaustive search is thus quickly rendered intractable. The observation that only a (very) small fraction of all combinations of fluxes explored are members of the MUST sets motivated the development of a direct enumeration approach that circumvented the need to exhaustively assess all combinations. This underlying mathematical problem is similar to the one we faced before for the targeted enumeration of higher order synthetic lethals [45]. Therefore, we modified the developed bilevel optimization representation to track reaction combinations with membership in MUST sets as opposed to causing synthetic lethality.

We present the bilevel optimization formulation for the identification of the membership of the MUST^LU^ set. The corresponding optimization formulations for other higher-order MUST sets can be recapitulated in a straightforward fashion. A particular reaction pair (*j*_1_, *j*_2_) belongs to MUST^LU^ if either flux *v_j_*_1_ must decrease or flux *v_j_*_2_ must increase to meet the imposed overproduction target. Mathematically, this implies satisfying the following inequality: . The ranges for difference in flux values for two reactions in the overproducing network falls completely to the left of the corresponding ranges for the wild-type network (i.e., less than ) only when the flux of reaction *j*_1_ decreases or the flux of reaction *j*_2_ increases in the face of overproduction. When searching for (*j*_1_, *j*_2_) pairs belonging to MUST^LU^, reactions *j*_1_ in MUST^L^ and *j*_2_ in MUST^U^ are first excluded from consideration. Binary variables $y_{1,j}^{L}$ and $y_{2,j}^{U}$ encode whether a particular reaction pair (*j*_1_, *j*_2_) belongs to MUST^LU^ by both assuming a value of one. The following bilevel formulation is solved iteratively using integer cuts to exclude the previously found solution to identify the complete membership of set MUST^LU^.

We recursively solve the optimization problem after employing integer cuts [68] until all the reaction pairs in MUST^LU^ are exhaustively enumerated.
