# Supplementary material for: OptForce: An Optimization Procedure for Identifying All Genetic Manipulations Leading to Targeted Overproductions
Source: PLoS Comput Biol. 2010 Apr 15;6(4):e1000744. doi: 10.1371/journal.pcbi.1000744 (PMC2855329; doi:10.1371/journal.pcbi.1000744)
Supplement: Text S3 — Appendix C: Bilevel formulation for the identifying the FORCE set (0.07 MB DOC) [file pcbi.1000744.s003.doc]

**OptFlux: An Optimization Procedure for Identifying All Genetic Manipulations Leading to Targeted Overproductions**

**Supporting Information: Text S3**

Sridhar Ranganathan^1^, Patrick F. Suthers^2^ and Costas D. Maranas^2^,*

**Appendix C: Bilevel formulation for the identifying the FORCE set**

The collection of MUST sets encode all combinations (and possibilities) of flux changes that must happen in the network in the face of an imposed overproduction target. They can be represented as a logic diagram containing AND and OR operators. However, not every required network flux change must be imparted through a genetic modification. All MUST set requirements can simultaneously be satisfied by imparting a much smaller number of genetic modifications due to reaction flux couplings through stoichiometry. We refer to the minimal number of such genetic manipulations as the FORCE set. As was the case with the MUST sets, there typically exists multiple ways of constructing the FORCE set leading again to a logic diagram representation of representing the totality of engineering manipulations leading to the satisfaction of an imposed overproduction target.

We introduce an optimization formulation for the enumeration of the FORCE set that identifies engineering modifications that satisfy the imposed overproduction target for any feasible flux combination in the network. This implies that unlike OptKnock results, the identified FORCE set is valid irrespective of whether maximization of biomass or any other objective function drives flux allocation in the network. This conservative posture implies that it is possible that the overproduction target could be met after only a subset of the engineering modification in the FORCE set are imposed. The optimization formulation is a bilevel problem accounting for three different types of direct engineering interventions leading to an increase, decrease or elimination of a reaction flux. Sets M*^U^*, M*^L^* and M× contain the candidate reactions from the MUST sets that can be forced up, down or off, respectively to pursue the imposed overproduction target(s). Three separate sets of binary variables are defined to model these choices:

The proposed optimization formulation can be expressed as follows:

The inner optimization problem finds the “worse-case” scenario for the production of the targeted product while the outer part aims to meet the overproduction target (even for the worst case scenario) with the minimum number of engineering modifications. Constraints (16-18) propagate the effect of engineering modifications encoded within to the corresponding reaction fluxes. For example, if a reaction is selected for up regulation then the flux for this reaction is set greater than the maximum value allowed in the wild-type strain ($v_{j}^{\text{max}}$). Subsequently, we employ integer cuts and identify all the alternate solutions for *k*-interventions that guarantee the target yield for the product.
