# Supplementary material for: OptForce: An Optimization Procedure for Identifying All Genetic Manipulations Leading to Targeted Overproductions
Source: PLoS Comput Biol. 2010 Apr 15;6(4):e1000744. doi: 10.1371/journal.pcbi.1000744 (PMC2855329; doi:10.1371/journal.pcbi.1000744)
Supplement: Text S4 — Results for MUST considered four-at-a-time (quadruples) (0.12 MB DOC) [file pcbi.1000744.s004.doc]

**OptFlux: An Optimization Procedure for Identifying All Genetic Manipulations Leading to Targeted Overproductions**

**Supporting Information: Text S4**

Sridhar Ranganathan^1^, Patrick F. Suthers^2^ and Costas D. Maranas^2^,*

**Results for MUST considered four-at-a-time (quadruples):**

Case 1: Succinate overproduction target at 100% of its theoretical maximum yield

| **MUST^UUUU^** | **ATPS4rpp** | **ACALD** | **CS** | **ENO** |
| --- | --- | --- | --- | --- |
| **MUST^uuul^** | **ATPS4rpp** | **MALS** | **ACALD** | **PFL** |
|  | **ACS** | **ACALD** | **PPC** | **PTAr** |
|  | **ATPS4rpp** | **ASPT** | **PPC** | **FUM** |
|  | **GND** | **GLNS** | **CS** | **GLUDy** |
|  | **IMPC** | **GART** | **PPC** | **PFL** |
| **MUST^uull^** | **ACONTa** | **AICART** | **FUM** | **ASPTA** |
|  | **ACONTb** | **MALS** | **MDH** | **ASPTA** |
|  | **MALS** | **ATPS4rpp** | **PGK** | **PFL** |
|  | **ACONTb** | **GLNS** | **FUM** | **GLUDy** |
|  | **PPC** | **ACOTA** | **PFL** | **ASPTA** |
|  | **ACALD** | **ACS** | **AST** | **PTAr** |
|  | **ACKr** | **PTAr** | **TPI** | **PGK** |
|  | **CS** | **GLUSy** | **GLUDy** | **RPI** |
| **MUST^ulll^** | **ACONTa** | **AICART** | **FUM** | **ASPTA** |
|  | **ACONTb** | **MALS** | **MDH** | **ASPTA** |
|  | **MALS** | **ATPS4rpp** | **PGK** | **PFL** |
|  | **ACONTb** | **GLNS** | **FUM** | **GLUDy** |
|  | **PPC** | **ACOTA** | **PFL** | **ASPTA** |
|  | **ACALD** | **ACS** | **AST** | **PTAr** |
|  | **ACKr** | **PTAr** | **TPI** | **PGK** |
|  | **CS** | **GLUSy** | **GLUDy** | **RPI** |
| **MUST^llll^** | **PFL** | **TPI** | **ASPTA** | **PGK** |

The minimal set of network modifications are shown in Figure S6a.

Case 2: Succinate overproduction target at 98% theoretical yield while allowing for 1% yield of biomass:

| **MUST^uuuu^** | **ACALD** | **ATPS4rpp** | **PPC** | **CS** |
| --- | --- | --- | --- | --- |
|  | **ACKr** | **PPC** | **PTAr** | **MALS** |
|  | **G6PDH2r** | **AICART** | **ICL** | **PPC** |
|  | **IMPD** | **ACONTb** | **HXAND** | **PPC** |
|  | **RPI** | **GND** | **CS** | **PGM** |
|  | **PGM** | **TPI** | **PGK** | **ACONTb** |
|  | **PGM** | **GAPD** | **PPC** | **ACONTa** |
|  | **ACONTb** | **ATPS4rpp** | **ACONTa** | **PUNP2** |
| **MUST^uuul^** | **ACONTa** | **ACONTb** | **ATPS4rpp** | **2AGPA140tipp** |
|  | **ACALD** | **CS** | **ATPS4rpp** | **PGK** |
|  | **ACALD** | **ACS** | **PPC** | **PTAr** |
| **MUST^uull^** | **CS** | **MALS** | **MDH** | **ASPTA** |
|  | **AICART** | **ACONTa** | **FUM** | **ASPTA** |
|  | **GLUSy** | **ACONTb** | **FUM** | **GLUDy** |
|  | **ATPS4rpp** | **MALS** | **PGM** | **PFL** |
|  | **GAPD** | **PPC** | **TPI** | **RPI** |
| **MUST^ulll^** | **ACONTb** | **PGI** | **FUM** | **ASPTA** |
|  | **PPC** | **TPI** | **PGK** | **RPI** |

The results for the minimal set of network modifications are shown in Figure S6b.

Case 3: Succinate overproduction upon the addition of pyruvate carboxylase

| **MUST^uuuu^** | **ACS** | **GLCtex_f** | **ACKr_f** | **ACALD_f** |
| --- | --- | --- | --- | --- |
| **MUST^uuul^** | **ACONTa** | **AICART** | **FUM** | **ASPTA** |
| **MUST^uull^** | **PPC** | **ACOTA** | **PFL** | **ASPTA** |
|  | **ACALD** | **ACS** | **AST** | **PTAr** |
| **MUST^ulll^** | **PFL** | **TPI** | **ASPTA** | **PGK** |

The results for the minimal set of network modification are shown in Figure S6c.
