# Supplementary material for: OptForce: An Optimization Procedure for Identifying All Genetic Manipulations Leading to Targeted Overproductions
Source: PLoS Comput Biol. 2010 Apr 15;6(4):e1000744. doi: 10.1371/journal.pcbi.1000744 (PMC2855329; doi:10.1371/journal.pcbi.1000744)
Supplement: Text S5 — Prototype Implementation for the OptForce Algorithm (0.06 MB DOC) [file pcbi.1000744.s005.doc]

**OptFlux: An Optimization Procedure for Identifying All Genetic Manipulations Leading to Targeted Overproductions**

**Supporting Information: Text S5**

Sridhar Ranganathan^1^, Patrick F. Suthers^2^ and Costas D. Maranas^2^,*

**OptForce Algorithm:**

**Step 1:** Estimate the maximal flux variability for the wild-type (initial) strain (Use optimization formulations in S1)

**Step 2:** Identify the MUST set of reactions:

1. Identify the MUST set of reactions considered one-at-a-time (MUST^U^, MUST^L^, MUST^X^)
2. Identify the MUST set of reactions considering the sums and differences of two fluxes at a time (MUST^UU^, MUST^UL^, MUST^LL^) using optimization formulations shown in supplementary information (Text S2). Selectively remove reactions from the sets MUST^U^, MUST^L^ and MUST^X^ from appearing in the pairs by equating $y_{.,j}^{L}$ and $y_{.,j}^{U}$ to zeroes for these reactions.
3. Identify the MUST set of reactions considering the sums and difference of three fluxes at a time (MUST^UUU^, MUST^UUL^, MUST^ULL^ etc.). Selectively remove reactions from the MUST sets for singles and pairs by setting the binary variables to zero values.

**Step 3:** Identify the FORCE set of engineering interventions using optimization formulations described in Text S3.

**Sample GAMS Code:**

**** Outer Problem:

outer.. z =e= v('EX_succ(e)');

outer1.. sum(j, yu(j) + yl(j) + y0(j) ) =e= k;

outer2(j).. yu(j) + yl(j) + y0(j) =l= 1;

outer3.. v('EX_succ(e)') =e= -100*mu('EX_glc(e)') + minbio * mu('Ec_biomass_iAF1260_WT_59p81M') + sum(j, wtheta(j)*basemax(j) + theta(j)*LB(j) - wtheta(j)*LB(j) ) -

sum(j, wphi(j)*basemin(j) + phi(j)*UB(j) - wphi(j)*UB(j) ) +

sum(j, deltap(j)*LB(j) - deltam(j)*UB(j) );

**** Linearizing Constraints:

outer4(j).. wtheta(j) =l= bigM*yu(j);

outer5(j).. wtheta(j) =g= -bigM*yu(j);

outer6(j).. wtheta(j) =l= theta(j) + bigM*(1-yu(j) );

outer7(j).. wtheta(j) =g= theta(j) - bigM*(1-yu(j) );

outer8(j).. wphi(j) =l= bigM*yl(j);

outer9(j).. wphi(j) =g= -bigM*yl(j);

outer10(j).. wphi(j) =l= phi(j) + bigM*(1-yl(j) );

outer11(j).. wphi(j) =g= phi(j) - bigM*(1-yl(j) );

outer20.. sum(j$(not interventions(j)), yu(j) + yl(j) + y0(j) ) =e= 0;

outer21(j)$(not mustu(j)).. yu(j) =e= 0;

outer22(j)$(not mustl(j)).. yl(j) =e= 0;

outer23(j).. y0(j) =e= 0;

primal.. zprimal =e= v('EX_succ(e)');

primal1(i).. sum(j, (S(i,j)*v(j))) =e= 0;

primal2.. v('EX_glc(e)') =e= -100;

primal3(j)$(mustu(j)).. v(j) =g= basemax(j)*yu(j) + LB(j)*(1-yu(j) );

primal4(j)$(mustl(j)).. v(j) =l= basemin(j)*yl(j) + UB(j)*(1-yl(j) );

primal5(j)$(must0(j)).. v(j) =g= LB(j)*(1-y0(j) );

primal6(j)$(must0(j)).. v(j) =l= UB(j)*(1-y0(j) );

primal7.. v('Ec_biomass_iAF1260_WT_59p81M') =e= minbio;

dual.. zdual =e= -100*mu('EX_glc(e)') + minbio*mu('Ec_biomass_iAF1260_WT_59p81M') +

sum(j, theta(j)*basemax(j)*yu(j) + theta(j)*LB(j) - theta(j)*LB(j)*yu(j) ) -

sum(j, phi(j)*basemin(j)*yl(j) + phi(j)*UB(j) - phi(j)*UB(j)*yl(j) ) +

sum(j, deltap(j)*LB(j)*(1-y0(j) ) - deltam(j)*UB(j)*(1-y0(j) ) );

dual1.. sum(i, lambda(i)*S(i, 'EX_succ(e)')) + theta('EX_succ(e)') –

phi('EX_succ(e)') + deltap('EX_succ(e)') - deltam('EX_succ(e)') =e= 1;

dual2.. sum(i, lambda(i)*S(i, 'EX_glc(e)')) + mu('EX_glc(e)') +

theta('EX_glc(e)') - phi('EX_glc(e)') + deltap('EX_glc(e)') - deltam('EX_glc(e)') =e= 0;

dual3(j)$(not constraint(j) ).. sum(i, lambda(i)*S(i,j) ) + theta(j) - phi(j) + deltap(j) - deltam(j) =e= 0;

dual4.. sum(i, lambda(i)*S(i, 'Ec_biomass_iAF1260_WT_59p81M')) +

mu('Ec_biomass_iAF1260_WT_59p81M') + theta('Ec_biomass_iAF1260_WT_59p81M') - phi('Ec_biomass_iAF1260_WT_59p81M') + deltap('Ec_biomass_iAF1260_WT_59p81M') - deltam('Ec_biomass_iAF1260_WT_59p81M') =e= 0;
