# Supplementary figures and images for: OptForce: An Optimization Procedure for Identifying All Genetic Manipulations Leading to Targeted Overproductions
Source: PLoS Comput Biol. 2010 Apr 15;6(4):e1000744. doi: 10.1371/journal.pcbi.1000744 (PMC2855329; doi:10.1371/journal.pcbi.1000744)

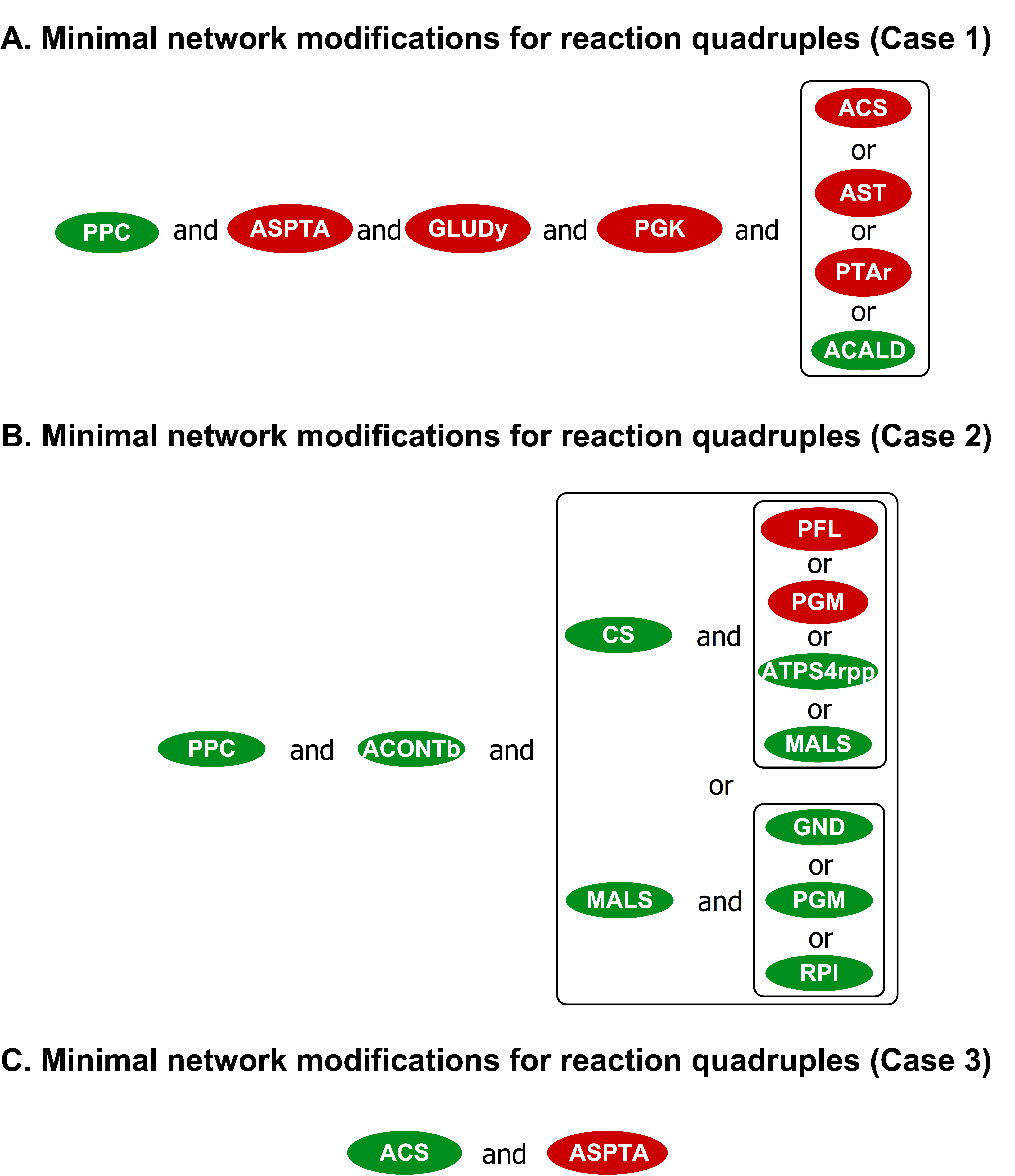

Supplement: Figure S1 — Minimal set of network modifications for reaction quadruples. (2.08 MB TIF) [file pcbi.1000744.s006.tif]

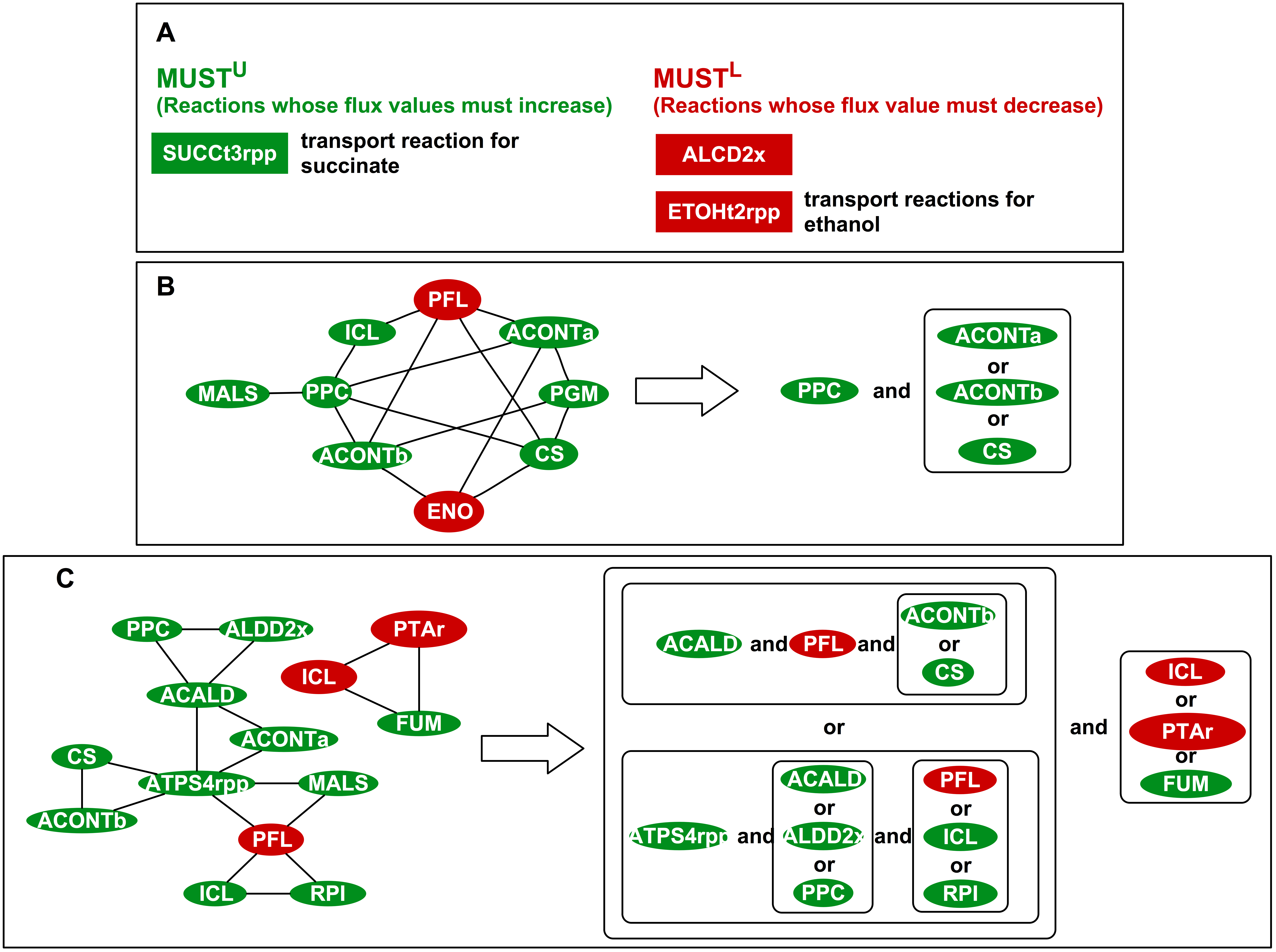

Supplement: Figure S2 — MUST set of reactions for 98% yield of succinate. Figure S2a shows the list of reactions in the MUSTU and MUSTL sets. Figure S2b and S2c shows the network of interacting reactions and the minimal set of network modification for the doubles and triples, respectively, identified for case 2. (3.34 MB TIF) [file pcbi.1000744.s007.tif]
